# Remodeling of the terpenoid metabolism during prolonged phosphate depletion in the marine diatom *Phaeodactylum tricornutum*

**DOI:** 10.1111/jpy.70014

**Published:** 2025-04-15

**Authors:** Florian Pruckner, Luca Morelli, Payal Patwari, Michele Fabris

**Affiliations:** ^1^ SDU Biotechnology, Department of Green Technology University of Southern Denmark Odense M Denmark; ^2^ SDU Climate Cluster, Faculty of Science University of Southern Denmark Odense M Denmark

**Keywords:** carotenoid biosynthesis, diatoms, geranyl diphosphate, isoprenoid metabolism, *Phaeodactylum tricornutum*, phosphate depletion, sterol biosynthesis

## Abstract

Terpenoids are a diverse class of naturally occurring organic compounds, which derive from five‐carbon isoprene units and play crucial roles in physiology, ecological interactions such as defense mechanisms, or adaptation to environmental stresses. In *Phaeodactylum tricornutum*, some of the most important isoprenoids are sterols and pigments, derived from precursors of the cytosolic mevalonate and the plastidial methyl‐erythritol 4‐phosphate pathway, respectively. However, the regulation of isoprenoid metabolism in *P. tricornutum* has not yet been characterized, presenting a major gap in our understanding of its ecological functions and adaptations. By leveraging metabolic, photosynthetic, and transcriptomic analyses, we characterized the dynamic remodeling of the isoprenoid pathways during prolonged nutrient stress in wild‐type diatoms. We observed the down‐regulation of the methylerythritol 4‐phosphate and pigment biosynthesis pathways and the upregulation of key genes in the mevalonate and sterol biosynthesis pathways. At the metabolite level, we observed an overall decrease in pigment and no changes in sterol levels. Using a genetically engineered diatom strain to produce a heterologous monoterpenoid to monitor the availability of one of the main terpenoid precursors, geranyl diphosphate (GPP), we suggest that cytosolic GPP pools increase during prolonged phosphate depletion. Our results have demonstrated how the biosynthesis of isoprenoid metabolites and the pools of prenyl phosphate are vastly remodeled during phosphate depletion. We anticipate that the knowledge generated in this study can serve as a foundation for understanding ecological responses and adaptations of diatoms to nutrient stress, contributing to our broader comprehension of marine ecosystem dynamics and design strategies for producing high‐value compounds in diatoms.

**Abbreviations**
AltSQE: alternative squalene epoxidase
CB: Calvin–Benson (cycle)
DEG: differentially expressed gene
DHAP: dihydroxyacetone phosphate
DMAPP: dimethylallyl diphosphate
DXR: 1‐deoxy‐d‐xylulose‐5‐phosphate reductoisomerase
ESAW: enriched seawater artificial water
FC: fold change
FDR: false discovery rate
FID: flame ionization detector
FPP: farnesyl diphosphate
FPPS: farnesyl diphosphate synthase
*F*_v_/*F*_m_
: maximum quantum yield of photosystem II
G3P: glyceraldehyde 3‐phosphate
GES: geraniol synthase
GGPP: geranylgeranyl diphosphate
GGPPS: geranylgeranyl diphosphate synthase
GO: to gene orthology
GPP: geranyl diphosphate
GPPS: geranyl diphosphate synthase
HAB: harmful algal bloom
HMGS: 3‐hydroxy‐3‐methyl‐glutaryl‐CoA synthase
HSF1/HSF3: heat shock factors 1 and 3
IDI: isopentenyl‐diphosphate delta isomerase
IDI‐SQS: isopentenyl diphosphate isomerase‐squalene synthase
IPK: isopentenyl phosphate kinase
IPM: isopropyl myristate
IPP: isopentenyl diphosphate
MCT: 2‐C‐methyl‐d‐erythritol 4‐phosphate cytidylyltransferase
MEP: methylerythritol 4‐phosphate
MVA: mevalonate
MVK: mevalonate kinase
OJIP: origin, J‐step, I‐step, and peak (fluorescence phases)
OSC: oxidosqualene cyclase
PAM: pulse amplitude modulated
PBS: phosphate‐buffered saline
PCA: principal component analysis
PHR: phosphate starvation response regulator
PSDP: presqualene diphosphate
PSII: photosystem II
PSY: phytoene synthase
PtIDISQS: isopentenyl diphosphate isomerase squalene synthase (*P. tricornutum* fusion protein)
PtPSR: phosphate starvation response (*P. tricornutum*)
PtTryp2: trypsin‐like protease in *P. tricornutum*
SPX: SPX domain‐containing protein
SREBP: sterol‐responsive element‐binding protein
TAGs: triacylglycerols
TF: transcription factor
uLoop: universal loop assembly
Vtc4: vacuolar transport chaperone 4
Vtp1: vacuolar phosphate transporter protein 1

## INTRODUCTION

Diatoms are among the most abundant groups of phytoplankton observed in nearly all aquatic habitats on Earth (Serôdio & Lavaud, 2022). They rapidly adapt to changing conditions, often outcompeting other autotrophs in phytoplankton blooms (Kuhlisch et al., 2024). A relevant portion of their metabolism is dedicated to the biosynthesis of terpenoids, which also play a central role in their adaptability (Lavaud et al., 2002). These compounds include various primary and specialized metabolites, which perform various essential functions, such as chemical defense (Brunson et al., 2018); cell membrane dynamics regulatory (Fabris et al., 2014); antioxidant, cell signaling, and regulation (Gallo et al., 2017); and photosynthetic. Furthermore, diatoms often form blooms and can produce harmful terpenoid‐based toxins, such as domoic acid (Brunson et al., 2018). These harmful algal blooms (HABs) can remodel the species balance in ecosystems, cause huge financial losses to fishing industries, and pose a threat to human health (Yan et al., 2022; Young et al., 2020). There is also evidence that sterol sulfates, produced by diatoms during blooms, can influence their growth and can trigger programmed cell death in the late stages of algal blooms (Gallo et al., 2017; Nuzzo et al., 2018). Apart from pigments, the role of other terpenoids in phytoplankton has not been thoroughly investigated. Generally, the major metabolite classes of isoprenoids in *Phaeodactylum tricornutum* comprise sterols and carotenoids (Fabris et al., 2020) that are likely synthesized in the endoplasmic reticulum and the chloroplast, respectively (Cvejić & Rohmer, 2000). Whereas research on *P. tricornutum* has shown how different culturing conditions, mostly distinguished by different light regimes, influence the accumulation of pigments (Ding et al., 2023; Truong et al., 2023), little is known about how environmental conditions influence the productivity and output of the mevalonate (MVA) and sterol biosynthesis pathways (Jaramillo‐Madrid et al., 2019).

*Phaeodactylum tricornutum* synthesizes the precursors for terpenoid biosynthesis, isopentenyl diphosphate (IPP) and dimethylallyl diphosphate (DMAPP), via two distinct pathways: the cytosolic MVA pathway and the plastidial methylerythritol 4‐phosphate (MEP) pathway. Generally, the MVA pathway provides precursors for sterol and the MEP pathway for pigment biosynthesis (Cvejić & Rohmer, 2000). Dimethylallyl diphoshate and IPP are subsequently condensed step by step to form longer chains by prenyltransferases. It is presumed that in the cytosol, the enzyme geranyl diphosphate (GPP) synthase (GPPS) condenses two units of DMAPP to form GPP. Geranyl diphosphate and an additional DMAPP are condensed by farnesyl diphosphate synthase to form farnesyl diphosphate (FPP; Fabris et al., 2014). Two units of FPP finally are condensed by the fusion protein isopentenyl diphosphate isomerase squalene synthase (PtIDISQS; Phatr3_EG02290) to form the 30C squalene with presqualene diphosphate (PSDP) as an intermediate step (Fabris et al., 2014). In the plastid, it is presumed that DMAPP and IPP also are converted into GPP by a GPPS, with two GPP units subsequently being condensed by a geranylgeranyl diphosphate synthase (GGPPS) into geranylgeranyl diphosphate (GGPP). Geranylgeranyl diphosphate is finally converted into phytoene via phytoene synthase (PSY; Phatr3_EG02349; Dambek et al., 2012). The content and diversity of sterols vary greatly from diatoms to organisms from different taxa, such as plants, fungi, or animals. Diatoms have unique metabolic features in their isoprenoid and sterol biosynthesis pathways, combining aspects from plants, fungi, and animals (Fabris et al., 2014). *Phaeodactylum tricornutum* accumulates free pools of GPP (Fabris et al., 2020), and its sterol metabolism is characterized by the presence of atypical enzymes, such as an alternative squalene epoxidase (AltSQE, Phatr3_J45494; Pollier et al., 2019), a multifunctional isopentenyl diphosphate isomerase and squalene synthase (Phatr3_EG02290), and an extended oxidosqualene cyclase (Phatr3_EG02293; Fabris et al., 2014). Although most enzymatic steps in the biosynthesis of isoprenoids are mapped, the regulation of this portion of diatom metabolism and its dynamics in response to intracellular and environmental stimuli are almost entirely unknown. Understanding the role of diatom and phytoplankton terpenoids in a biological and ecological context will help us to understand better not only the diatom dominance and dynamics in current and future aquatic habitats but also how to cope with HABs more effectively.

More broadly, terpenoids comprise one of the largest classes of secondary metabolites, many of which have high industrial and commercial relevance, including carvacrol, citronellal, geraniol, linalool, menthol, and thymol (Hyldgaard et al., 2012). Photoautotrophic microorganisms, despite their naturally high terpenoid content and diversity (Pichersky & Raguso, 2018), are underutilized in the biotech industry due to lower yields compared to traditional heterotrophic organisms like *Escherichia coli* and *Saccharomyces cerevisiae* (Zhang & Hong, 2020). To establish photoautotrophs as a sustainable production platform, yields must be improved through strain optimization and culturing condition improvements. In the case of terpenoid production, this requires a deeper understanding of how phytoplankton regulate their terpenoid metabolic networks under changing environmental conditions.

How the limitation of nutrients such as nitrogen and phosphate can reprogram the metabolism of *Phaeodactylum tricornutum* to accumulate lipids has been widely investigated (Breuer et al., 2012; Cruz de Carvalho et al., 2016; Huang et al., 2019; Murison et al., 2023; Valenzuela et al., 2013; Wang et al., 2009; Zhang et al., 2021). A major transcription factor (TF) that regulates the response to phosphate depletion has been discovered in *P. tricornutum* and defined as a “phosphate starvation response” (PtPSR), also referred to as a PtPSR regulator (PHR, Phatr3_J47256; Kumar Sharma et al., 2020, p. 2380). In the presence of phosphate, PtPSR is bound and inhibited by SPX (Phatr3_J47434; K. Zhang et al., 2021), which gets degraded by the protease PtTryp2 (Phatr3_J54319) when N:P ratios increase (You et al., 2022). Although the impact of short‐term phosphate starvation on *P. tricornutum* has been well researched, there is limited understanding of its effects over longer periods. That is, unlike short‐term nutrient stress, the consequences of prolonged phosphate depletion remain underexplored. This is particularly relevant, as phytoplankton often experience seasonal fluctuations in nutrient availability, driven by ocean currents, requiring them to endure phosphate‐limiting conditions for weeks or even months at a time (Arteaga et al., 2014). Cruz de Carvalho et al. (2016) investigated the effects of phosphate depletion after 8 days. Their work revealed the upregulation of genes associated with catabolic functions such as glycolysis, carbohydrate catabolic processes, and phosphate transporters, whereas down‐regulated genes were mostly associated with photosynthesis and, notably, isoprenoid metabolism and lipid biosynthetic processes.

Cruz de Carvalho et al.'s (2016) work sparked the research questions and served to design the experimental setup of our study. By mining the dataset of Cruz de Carvalho et al. on DiatOmicBase (Villar et al., 2024), specifically focusing on the terpenoid metabolic network of *Phaeodactylum tricornutum*, which was unexplored by the authors, we observed a general upregulation of genes associated with the MVA and sterol biosynthesis pathways. As this, to the best of our knowledge, represented the first instance in which upregulation of these pathways was observed, we sought to fully characterize this condition and its effects on terpenoid metabolism regulation. In this work, we have comprehensively profiled the terpenoid metabolic network of *P. tricornutum* and the transcriptional and biochemical levels under prolonged phosphate depletion. We have provided a detailed overview of how this condition affects sterol and pigment accumulation, photosynthetic activity, and the rebalancing of DMAPP and GPP metabolite pools.

## MATERIALS AND METHODS

### Culturing conditions and growth monitoring

*Phaeodactylum tricornutum* (CCMP632, CCAP 1055/1) was cultured in enriched seawater artificial water (ESAW) medium (Berges et al., 2001) under a 12:12 h light:dark photoperiod at an irradiance of 90 μmol photons · m^−2^ · s^−1^. Cultures were maintained at 21°C in an Innova S44i light incubator (Eppendorf, Germany) with continuous shaking at 95 rpm. Growth was monitored either by measuring optical density using a Shimadzu UV‐1280 spectrophotometer or by cell count using a Guava H5 flow cytometer (Cytek, Japan).

For phosphate depletion experiments, cells of a phosphate‐repleted culture were pelleted by centrifugation at 4000 rcf for 5 min, washed twice with ESAW without phosphate, and used to inoculate a culture with ESAW without phosphate with a cell density of 0.25 × 10^6^ cells · mL^−1^. Phosphate‐depleted cultures were cultivated over 8 days. Cultures were placed in the center of the incubator, and their respective positions were randomly changed every second day.

### Photosynthetic measurements

For biooptical assessments, 2 mL of each replicate culture was diluted with 2 mL of ESAW to reduce the risk of signal saturation. Chlorophyll *a* (chl *a*) fluorescence was measured using a pulse‐amplitude‐modulated (PAM) fluorometer (AquaPen‐C, AP 110‐C, Photon System Instruments, Czech Republic). Chlorophyll (chl) transient light curves were generated using the preprogrammed origin, J‐step, I‐step, and peak (fluorescence phases) (OJIP) protocol, which captures fluorescence rise through the four phases: O, J, I, and P (origin, J‐step, I‐step, and peak; Solovchenko et al., 2013).

### Pigments' extraction and analysis

After 8 days of culturing and 4 h after the onset of the light period, 50 mL of late exponential cultures of *Phaeodactylum tricornutum* cultured in ESAW with or without phosphate were harvested by centrifugation at 4000 rcf for 5 min, washed once with phosphate‐buffered saline (PBS), resuspended, and transferred into a 1.5‐mL microcentrifuge tube, pelleted again by centrifugation at 13,000 rcf, flash‐frozen in liquid nitrogen (after removal of the supernatant), and stored at −80°C. The 1.5‐mL microcentrifuge tube was weighed before the material was transferred into it.

The biomass was freeze‐dried in a Martin Christ (DE) ALPHA 1–2 LDplus (101521) freeze dryer. The samples were kept in a metal block precooled in liquid nitrogen to keep them frozen while drying. The weight of the cell pellets was determined by comparing the weight of the empty tube (before freeze‐drying) and the weight of the same tube containing the freeze‐dried pellet. The extraction was carried out by readapting the protocol by Mendes et al. (2007). Briefly, 1.5 mL of 95% (v/v) methanol buffered with 2% ammonium acetate (w/v) was added to the freeze‐dried pellet. The pellet was resuspended by vigorous vortexing and incubated for 3 h at 4°C in the dark on a spinner wheel. The extraction mixture was then centrifuged at 1100 rcf for 5 min at −4°C. The supernatant was filtered using 0.2‐μm pore size filters and injected into dark gas chromatography (GC) / high‐performance liquid chromatography (HPLC) glass sample vials. Analysis and quantification were performed on an Agilent (United States) 1200 Series HPLC instrument, which included a G1329A autosampler, a G1330B thermostat, a G1316A column compartment, an Agilent 1260 Infinity diode array detector (DAD) (G1315D), and a YMC Carotenoid column (CT99S03‐1503WT). The samples were analyzed with a two‐solvent system: solvent A (40% acetone/60% methanol) and solvent B (40% water/60% acetone). The flow rate was set at 0.05 mL · min^−1^, and the injection volume was 5 μL. The column temperature was maintained at 40°C. The HPLC method involved the following solvent gradient: starting with 40% solvent A, ramping to 70% solvent A over 3 min, and holding this ratio until minute 22. Subsequently, solvent A was ramped to 90% over 4 min and held for 5.5 min. Finally, solvent A was decreased to 60% over 3.5 min and maintained at this ratio, resulting in a total run time of 55 min. The absorbance of pigments was measured at 450 nm.

### Triterpenoids' extraction and analysis

After 8 days of culturing and 4 h after the onset of the light period, 50 mL of late exponential cultures of *Phaeodactylum tricornutum* cultured in ESAW with or without phosphate were harvested by centrifugation at 4000 rcf for 5 min, washed once with PBS, flash‐frozen in liquid nitrogen, and stored at −80°C. Before extraction, the samples were freeze‐dried. The weight of the cell pellets was determined by measuring the weight of the tube before and after freeze‐drying. To the freeze‐dried pellets, 250 μL of 40% (w/v) potassium hydroxide and 250 μL of 50% (v/v) ethanol were added. The pellets were resuspended by pipetting and incubated at 95°C for 10 min. Following this, 900 μL of hexane was added, and the mixture was incubated for 5 min at room temperature while being mixed on a rotor wheel. The organic (upper) phase was collected and stored in a GC/HPLC glass sample vial. This extraction with 900 μL of hexane was repeated, and the organic phases were pooled in the same vial. The hexane was evaporated by blowing nitrogen gas. To derivatize the sterols, 20 μL of pyridine and 100 μL of *N*‐methyl‐*N*‐trimethylsilyl‐trifluoroacetamide were added to the residue and incubated at 70°C for 1 h. The samples were analyzed using an Agilent 7890B gas chromatograph equipped with an Agilent J&W DB‐5 GC Column (122–5032), a flame ionization detector (FID), and an Agilent 7650A automatic liquid sampler. The inlet settings were an injection volume of 5 μL, an inlet heater temperature of 300°C, an inlet pressure of 11.22 psi, a septum purge flow of 3 mL · min^−1^, and a pulsed splitless injection with injection pulse pressure of 30 psi until 0.75 min. The column was maintained at a flow rate of 0.75 mL · min^−1^ with a pressure of 11.22 psi. The oven temperature program started at 90°C for 1 min, ramped to 275°C at a rate of 2.5°C · min^−1^, then ramped to 325°C at a rate of 30°C per min, and held for 5 min. The detector settings were a heater temperature of 365°C, an airflow of 300 mL · min^−1^, a hydrogen fuel flow of 30 mL · min^−1^, and a makeup flow of nitrogen at 20 mL · min^−1^.

### Generation of transgenic *Phaeodactylum tricornutum* cell lines

Episomes were assembled based on the universal loop assembly (uLoop) system (Pollak et al., 2020). Geraniol synthase (GES) from *Catharanthus roseus* was codon optimized for *Phaeodactylum tricornutum*, synthesized by Genewiz (Germany), and cloned into a pL0R uLoop plasmid as a “CD”‐uLoop part. It was further assembled with the uLoop parts *Phatr3_J49202* promoter (“AC”), 3x Stop codon (“DE”), and *Phatr3_J25172* (FcpB) terminator (“EF”) into a pCAo‐1 receiver plasmid. The assembled plasmid was cloned with a pCAo‐2 plasmid carrying a Zeocin resistance gene (Sh ble) and CEN‐ARS‐HIS region, and pCAo‐3 and pCAo‐4 spacer plasmids into a pCAe‐1 receiver plasmid. Episomes were introduced into *P. tricornutum* via bacterial conjugation with a protocol adapted from Karas et al. (2015). An exponentially growing *P. tricornutum* culture was used to inoculate a 50‐mL culture with a starting optical density at 750 nm (OD_750_) of ~0.03. The culture was grown until it reached an OD_750_ of 0.3. Six‐well plates were prepared to contain ~3.5 mL of ½ ESAW–5% luria bertani (LB)–1% agar per well and left under the laminar flow hood for 1 h to dry completely. The day before conjugation, *P. tricornutum* cultures in the mid to late exponential growth stage (OD_750_ = 0.3) were centrifuged at 3000 × *g*. For every 50 mL of harvested culture, 500 μL of ESAW was added, and 50 μL of this mixture was spotted onto the six‐well ½ESAW–5%LB agar plates. The plates were allowed to dry in a laminar flow hood until no visible liquid remained. Plates were then incubated under continuous light for ~18 h at 90 μmol photons · m^−2^ · s^−1^ at 21°C.

An overnight culture of an *Escherichia coli* Epi300 strain containing the conjugative plasmid (pTA‐MOB) and the cargo plasmid (episome) was started the night before the conjugation experiment (shaking at 180 rpm, 37°C). The overnight culture was used to inoculate 20 mL of fresh *E. coli* culture with a 1:50 dilution in LB supplemented with gentamycin 20 μg · mL^−1^ and spectinomycin 50 μg · mL^−1^. The flasks were shaken at 200 rpm and 37°C until the culture reached an OD_600_ of 0.8–1.0. The culture was then spun down for 10 min at 3000 × *g*. All supernatant was removed, and the cell pellet was gently resuspended in 250 μL of super optimal broth with catabolite repression (SOC) medium. Fifty microliters of the *E. coli* culture were pipetted onto the dried diatom spot in the multi‐well plate. The spot was not spread but allowed to dry under laminar flow. The plates were incubated for 90 min at 30°C in the dark and then moved to diatom growth conditions for a 3‐day recovery period. After 3 days, 1 mL of ESAW medium was added to the ½ESAW–5%LB plates. Cells were resuspended and transferred to a ½ESAW–agar plate with 100 μg · mL^−1^ zeocin and a suitable antibiotic against bacteria to select for transformed diatoms. Colonies of *Phaeodactylum tricornutum* were picked after 2–3 weeks using a sterile pipette tip and resuspended in 200 μL ESAW and 100 μg · mL^−1^ zeocin medium in 96‐well plates. These cultures were grown until a change in density indicated cell growth. The cultures were then inoculated into fresh 96‐well plates and screened on a Guava easyCyte HT (CyTek Biosciences) flow cytometer (excitation with a 488 nm laser and detection using a fluorescein isothiocyanate detector); transgenic diatom lines exhibiting the highest fluorescence were selected for experiments.

### RNA extraction and genome‐wide gene expression analysis

After 8 days of culturing and 4 h after the onset of the light period, 30 mL of *Phaeodactylum tricornutum* cultures were harvested by centrifugation for 1 min at 4000 × *g* at −9°C. The supernatant was discarded, and the pellet was quickly resuspended in ~1 mL of the remaining supernatant. This was transferred to a 2‐mL RNase‐free microcentrifuge tube (AM12475, Thermo Fisher Scientific, United States) and centrifuged for 15 s at 16,000 × *g*. The supernatant was discarded, and the microcentrifuge tube was flash‐frozen in liquid nitrogen and stored at −80°C. The harvesting process took no longer than 2–3 min, and RNA was extracted within 2 weeks of sampling.

For RNA extraction, the frozen pellet was resuspended in 1.5 mL of PureZol RNA isolation reagent (Cat. #7326890, Bio‐Rad, United States) by pipetting up and down and then incubated at room temperature for 5 min. Following this, 300 μL of chloroform were added, and the mixture was incubated for an additional 15 min at room temperature with occasional mixing by inversion. The suspension was then centrifuged at 12,000 rcf for 15 min at 4°C. A volume of 650 μL of the upper phase was transferred to a fresh RNase‐free microcentrifuge tube, to which an equal volume of 75% ethanol was added and mixed thoroughly by pipetting. The mixture was then transferred to a RNeasy Mini Kit column from the Qiagen RNeasy Mini Kit (74,106, QIAGEN, Netherlands) and processed according to the manufacturer's instructions.

RNA quantity was measured using a NanoPhotometer N50/N60 (Techno Scientific, United StatesUSA), and RNA purity and degradation were assessed on a 1% agarose gel. Library preparation was performed using the NEBNext Ultra II RNA Library Prep Kit (E7770L, NEB, United States). The quality of the prepared libraries was assessed using a Fragment Analyzer, and sequencing was conducted on the NovaSeq 6000 platform (Illumina).

The quality of reads was assessed before and after trimming with Trim Galore (Krueger, 2016/Krueger, 2024) using FastQC (Andrews, 2017/Andrews, 2024) with default settings. Reads were mapped to the reference genome ASM15095v2 (RefSeq accession: GCF_000150955.2) of *Phaeodactylum tricornutum* using HiSAT2 (Deahwan, 2015/Deahwan, 2024). Quantitation was performed in SeqMonk (Babraham Bioinformatics, 2007). Duplicates were not removed, and default import settings for BAM files were used. Differential expression analysis was performed using DESeq2 after Benjamini and Hochberg correction. Quantitation was conducted using the RNA‐Seq pipeline quantitation on merged transcripts, counting reads over exons as raw counts, assuming a nonstrand‐specific library. Differential expression was considered significant with an adjusted *p*‐value below 0.01 and a log2 fold change above 1.5.

Gene orthology analysis was performed using g:profiler (Kolberg et al., 2023), employing Benjamini–Hochberg false discovery rate with a significance threshold of 0.05.

### Geraniol extraction and analysis

Since several independent *Phaeodactylum tricornutum* cell lines expressing the *Phatr3_J49202p_CrGES‐mVenus_ Phatr3_J25172t* on extrachromosomal episomes were thoroughly profiled in Fabris et al. (2020) and showed consistent growth and geraniol production phenotypes, as opposed to negative controls that did not produce monoterpenoids; one cell line harboring such construct was selected for experiments in triplicate. Geraniol produced by cultivating 50 mL of transgenic cultures harboring the *pPTBR11_Phatr3_J49202p_CrGES‐mVenus* episome were sequestered with 1.6 mL of isopropyl myristate (IPM; A18111.AP, Sigma‐Aldrich, United States) that was added to the media at inoculation. Cultures were grown in the presence of 50 μg · mL^−1^ zeocin. On the last day of cultivation, IPM was removed, transferred into a dark GC/HPLC vial, and used directly for analysis. Geraniol was measured by an Agilent 7890B gas chromatograph equipped with an Agilent J&W DB‐5 GC Column (122‐5032), FID, and an Agilent 7650A automatic liquid sampler. The inlet settings were an injection volume of 1 μL, an inlet heater temperature of 280°C, an inlet pressure of 21.92 psi, a septum purge flow of 3 mL · min^−1^, and a split ratio of 2:1. The column was maintained at a flow rate of 2 mL · min^−1^ with a pressure of 21.92 psi. The oven temperature program was started at 80°C for 1 min, ramped to 200°C at a rate of 30°C min^−1^, held for 1 min, then ramped to 320°C at a rate of 40°C · min^−1^ and held for 1 min. The detector settings were a heater temperature of 280°C, an airflow of 400 mL · min^−1^, a hydrogen fuel flow of 30 mL · min^−1^, and a makeup flow of nitrogen at 25 mL · min^−1^.

## RESULTS AND DISCUSSION

By mining a previously published transcriptomics investigation on prolonged phosphate depletion in *Phaeodactylum tricornutum* (Cruz de Carvalho et al., 2016), we detected a vast remodeling of terpenoid‐related pathways after 8 days of phosphate starvation. This included a partial down‐regulation of the MEP pathway, a mix of up‐ and down‐regulation of genes involved in pigment biosynthesis, a partial upregulation of the MVA pathway, and an upregulation of sterol biosynthesis genes. As this stress condition holistically impacts the expression of terpenoid‐related genes, we hypothesize that *P. tricornutum* rebalances its terpenoid composition, from pigments toward sterols, during prolonged phosphate starvation.

To systematically profile the terpenoid metabolism response at the transcriptional and metabolic levels, we cultivated *Phaeodactylum tricornutum* in the presence or absence of phosphate for 8 days and subsequently analyzed differentially expressed genes (DEGs) and quantified sterol and pigment contents of cultures, as well as their photosynthetic activity. Furthermore, we assessed how this stress condition affected growth and photosynthetic efficiency.

### Effects of prolonged phosphate depletion on growth and photosynthetic efficiency

**FIGURE 1:**
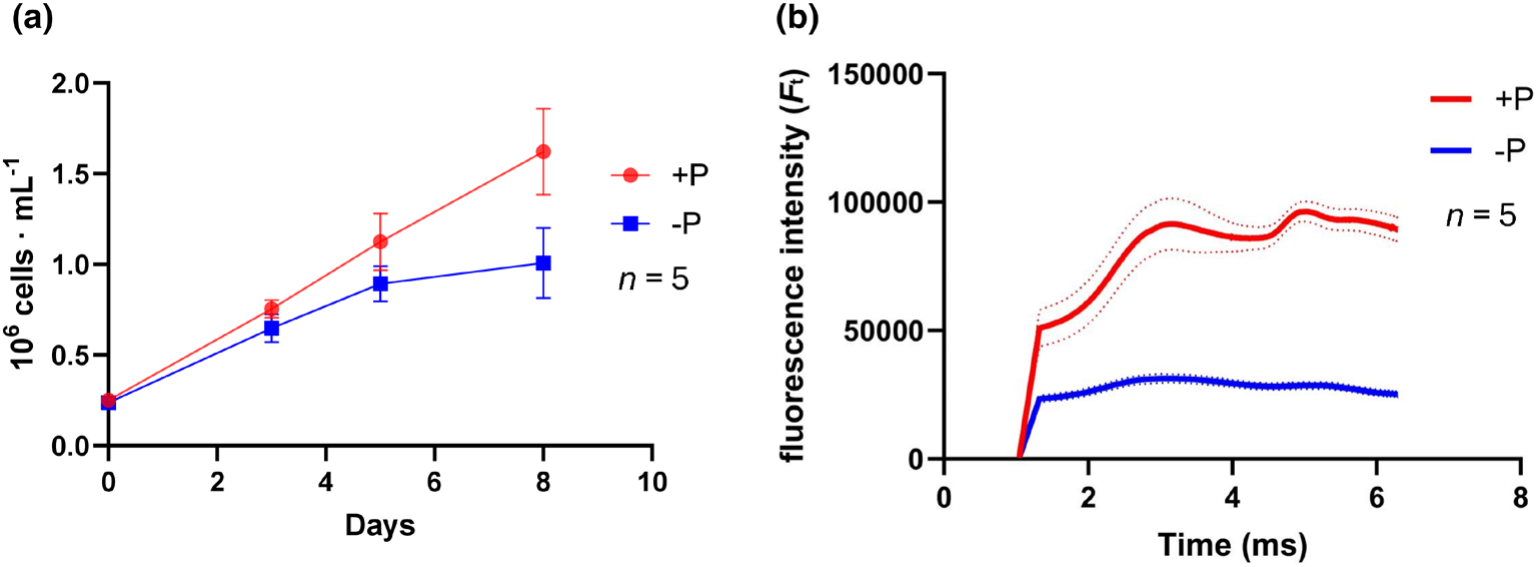
Physiological response of *Phaeodactylum tricornutum* to prolonged phosphorus depletion. (a) Growth curve of *P. tricornutum* over 8 days, comparing conditions with (+P) and without (−P) phosphorus. Cell density was determined by measurement of the optical density at 600 nm (OD_750nm_) and correlated to cell concentration. Error bars represent the standard deviation (*n* = 5). (b) origin, J‐step, I‐step, and peak (fluorescence phases) (OJIP) curve: This curve assesses the photosynthetic activity of 8‐day‐old cultures of *P. tricornutum* under phosphorus‐sufficient (+P) and phosphorus‐depleted (−P) conditions. The solid line represents the mean fluorescence intensity, while the dotted line indicates the standard deviation (*n* = 5).

Phosphate‐depleted cultures exhibited impaired growth and a paler color, consistent with previous studies on *Phaeodactylum tricornutum* under similar conditions (Alipanah et al., 2018; Figure 1a). Phosphorus is crucial for nucleic acids, phospholipids, and intermediate metabolites, and its availability affects primary production and the carbon cycle in aquatic environments. Additionally, phosphate‐starved cultures demonstrated decreased photosynthetic efficiency, as evidenced by the chl a transient fluorescence curve (OJIP transient; Figure 1b). The OJIP test analyzes the fate of photons absorbed by the photosystem II (PSII) antennae, and under optimal conditions, the curve is a sigmoid shape, indicating a high probability of exciton exchange between PSII units in a supercomplex (Antal et al., 2009).

In our study, only diatoms grown with sufficient phosphate availability showed active electron transport through the photosynthetic chain, while phosphate‐starved samples displayed a flat curve, indicating impaired photosynthetic activity. The deleterious effect of phosphate starvation on diatom photosynthetic efficiency, reflected in a reduced effective quantum yield of PSII (*F*v/*F*m), has been documented in other studies (Alipanah et al., 2018; Chai et al., 2023; Liu et al., 2011). This impairment is primarily due to the impact of phosphorus deficiency on the repair of photosystem proteins such as the D1 protein, which undergoes cycles of degradation and repair mediated by adenosine 5'‐triphosphate upon light exposure. Additionally, phosphorus depletion affects the expression of genes encoding key proteins like manganese‐stabilizing protein (PsbO), an extrinsic protein crucial for the oxygen‐evolving complex at the donor site of PSII, initiating the electron transport process (Popelkova & Yocum, 2011).

Notably, the growth of cultures with phosphate in the current study was not as fast as has been reported previously by Cruz de Carvalho et al. (2016). This is most likely due to the higher culture volumes used in this study (400 mL in contrast to 250 mL) and the consequent higher extent of self‐shadowing.

### Prolonged phosphate depletion induces differential gene expression in isoprenoid biosynthesis pathways

As the prolonged phosphate starvation transcriptomic data set of Cruz de Carvalho et al. (2016) was built with two replicates of each growth condition, we set out to investigate the perturbation of terpenoids and their related pathways with RNA‐seq data with higher statistical power. We therefore conducted our RNA‐Seq analysis using five replicates for each condition. Principal component analysis (PCA) and hierarchical clustering of samples showed clear similarities between replicates and distinctions between the different growth conditions (Figure S1). The PCA revealed that for the phosphate depleted condition, the replicates clustered within two distinct clusters (Figure S1b). Since clusters of the replicates of the two culturing conditions were distinguishable nonetheless, we decided to include all samples in the analysis. We could detect 6180 DEGs (3104 upregulated and 3076 downregulated; Wald test, Benjamin–Hochberg adjusted *p*‐value < 0.01). Due to our higher replicate number, we could detect more DEGs than in the data set of Cruz de Carvalho et al. (2016) when applying our analysis pipeline (3409 DEGs; 1869 upregulated and 1540 downregulated; Wald test, Benjamin–Hochberg adjusted *p*‐value < 0.01). Generally, the identified DEGs aligned well between the gene expression data sets of this study and the one from Cruz de Carvalho et al. (2016; Figure S2). However, a substantial number of genes in the Cruz de Carvalho et al. data set was not significantly differentially expressed in our experiment. The full list of DEGs is given in Table S1.

To evaluate the general functions associated with DEGs, we implied a log2 fold‐change (FC) threshold of 1.5, narrowing to 1466 DEGs (708 upregulated and 758 downregulated genes) and subjected these genes to gene orthology (GO) analysis. The GO analysis of these genes revealed that upregulated genes were enriched in their molecular function as phosphatases and TFs (Table S2). Notably, and as expected, the key phosphate stress regulators, *PtPSR* (*Phatr3_J47256*) and *PtTryp2* (*Phatr3_J54319*) both showed a log2FC of 2.59. *SPX* (*Phatr3_J47434*), which inhibits the function of PtPSR (Zhang et al., 2021), also showed a positive log2FC of 2.47, suggesting it may play a role in fine‐tuning the response to phosphate depletion. Downregulated genes were involved in other functions associated with photosynthesis, tetrapyrrole biosynthesis, and components of the light‐harvesting complexes (Table S2).

In the MEP pathway, we observed the downregulation of three out of 10 genes. These were 1‐deoxy‐d‐xylulose‐5‐phosphate reductoisomerase (*DXR*; *Phatr3_J9258*), 2‐c‐methyl‐d‐erythritol 4‐phosphate cytidylyltransferase (*MCT*; *Phatr3_J21829*), and isopentenyl‐diphosphate delta‐isomerase (*IDI*; *Phatr3_J12533*). DXR has been recognized in plants as a rate‐limiting step of the pathway (Movahedi et al., 2022). Even though not classically recognized as a rate‐limiting step of this pathway, the overexpression of MCT can facilitate a higher flux through the MEP pathway in plants (Jiang et al., 2024). The putatively plastidial IDI (*Phatr3_J12533*), which balances DMAPP and IPP levels, is also recognized as a rate‐limiting step of downstream pathway genes (Wang et al., 2019; Yahya et al., 2023).

**FIGURE 2:**
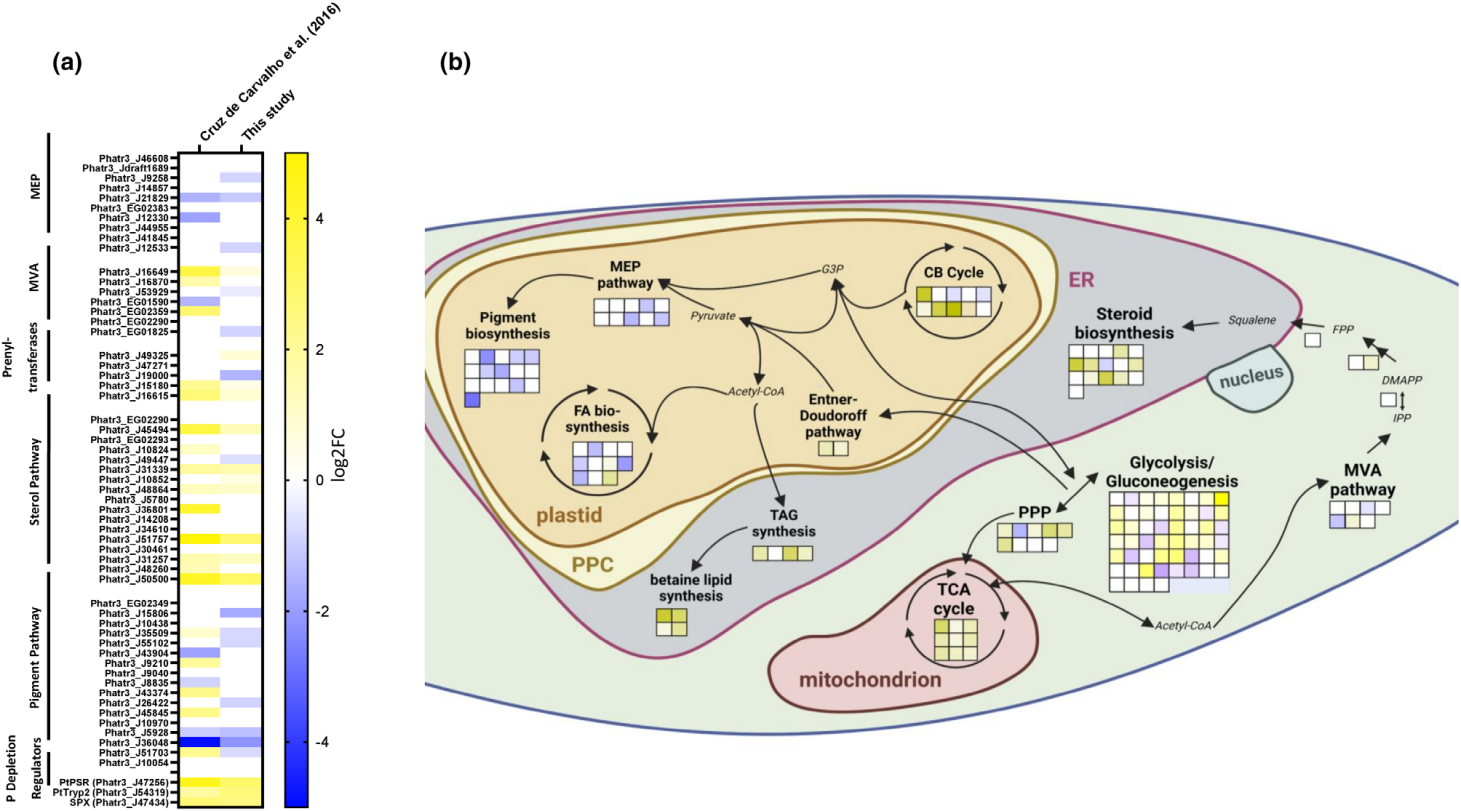
Analysis of differentially expressed genes (DEGs) in terpenoid biosynthesis and related pathways. (a) Comparison of the transcriptomic response of terpenoid‐related genes during prolonged phosphate starvation, referencing data from Cruz de Carvalho et al. (2016), alongside data from the current study. The three phosphate starvation regulators, *PtPSR*, *PtTryp2*, and *SPX*, are included as references for comparison between the data sets. (b) Visual representation of the differences in gene expression within various terpenoid pathways and related metabolic pathways based on the transcriptomics data obtained in this study. Glycolysis and gluconeogenesis are predicted to take place in several compartments, but for simplicity, it is represented only in the cytosol.

The MEP pathway synthesizes the precursors for the biosynthesis of carotenoids. In this pathway, seven out of 16 genes showed reduced expression, including phytoene desaturase‐like protein 1 (*Phatr3_J15806*), phytoene desaturase 1 (*Phatr3_J35509*), phytoene desaturase 2 (*Phatr3_J55102*), beta‐carotene hydroxylase (*Phatr3_J26422*), the precursor of protein zeaxanthin epoxidase‐like protein (*Phatr3_J5928*), the precursor of protein violaxanthin de‐epoxidase‐like protein (*Phatr3_J36048*), and violaxanthin de‐epoxidase (*Phatr3_J51703*). Phytoene desaturase is a key enzyme in the carotenoid biosynthesis pathway, which when knocked out in plants, leads to “albino” phenotypes and phytoene accumulation due to an inability to metabolize this intermediate metabolite toward carotenoids (Qin et al., 2007). The downregulation of phytoene desaturase genes could lead to a bottleneck in carotenoid biosynthesis, subsequently affecting the synthesis of downstream pigments such as fucoxanthin, β‐carotin, diatoxanthin, and diadinoxanthin. Therefore, this downregulation could disrupt the balance and flow of intermediates through the pathway, resulting in decreased levels of critical pigments that are essential for photoprotection and photosynthetic efficiency. Additionally, one of the two prenyltransferases predicted to be in the chloroplast, *Phatr3_J19000* (Fabris et al., 2020; Huang et al., 2024), presumably linking the MEP and the pigment biosynthesis pathway, was also downregulated (Figure 2). The combined downregulation of these key genes in the MEP pathway and subsequent pigment biosynthesis suggests a significantly lower flux of metabolites within these metabolic networks, potentially leading to a significant impact on the diatom's ability to cope with photooxidative stress and maintain optimal photosynthetic performance. The downregulation of pigment synthesis aligns well with existing data that have shown the downregulation of proteins associated with pigment binding and photosynthesis (Feng et al., 2015). This was also supported by the direct downregulation of pigments reported previously (Alipanah et al., 2018).

In the MVA pathway, we observed the upregulation of one and the downregulation of two genes out of seven. 3‐hydroxy‐3‐methyl‐glutaryl‐CoA synthase (*Phatr3_J16649*) was upregulated, possibly increasing the flux toward the production of MVA, a key intermediate in isoprenoid biosynthesis. Conversely, mevalonate kinase (*Phatr3_J53929*) and isopentenyl phosphate kinase (IPK) (*Phatr3_EG01825*) were downregulated, potentially limiting the conversion of MVA to downstream metabolites and affecting the overall flux of the pathway. Isopentenyl phosphate kinase has been suggested to be part of a two‐enzyme alternative side route through the MVA pathway (Dellas et al., 2013). However, the second enzyme of this pathway, mevalonate diphosphate decarboxylase (MDC; *Phatr3_EG02359*) was not differentially regulated. Overall, there was no clear trend in the up‐ or downregulation of the MVA pathway during prolonged phosphate depletion. The MVA directly provides building blocks for the biosynthesis of sterols in *Phaeodactylum tricornutum* (Cvejić & Rohmer, 2000; Fabris et al., 2014, 2020).

Interestingly, in the sterol biosynthesis pathway, we observed a more extensive transcriptional reprogramming. Although the expression of the genes involved in this pathway had been rarely perturbed in the environmental conditions studied so far and has been included in DiatomOmicBase (Villar et al., 2024), seven out of 17 genes were upregulated, while one gene was downregulated. The upregulated genes included key pathway nodes (Jaramillo‐Madrid et al., 2020): the first and rate‐limiting step by AltSQE (*Phatr3_J45494*), 14‐α‐demethylase (*Phatr3_J31339*), methylsterol monooxygenase (*Phatr3_J10852*), 3‐β‐hydroxysteroid‐4‐α‐carboxylate‐3‐dehydrogenase (*Phatr3_J48864*), delta14‐sterol reductase (*Phatr3_J31257*), putative 3‐beta‐hydroxy‐delta5‐steroid dehydrogenase (*Phatr3_J50500*), and the gene encoding the final step in the pathway, sterol C‐22 desaturase (*Phatr3_J51757*). This transcriptional upregulation suggests a robust increase in the synthesis of sterols. The single downregulated gene, cycloeucalenol cycloisomerase (*Phatr3_J49447*), showed only a small log2FC, which might indicate not so much a limiting step but a specific regulatory adjustment within the sterol biosynthesis pathway for balancing sterol intermediates and final products. The overall differential expressions of terpenoid‐related genes in this study and in that of Cruz de Carvalho et al. (2016) are summarized in Figure 2a.

Not only is the activity of a pathway regulated by its transcriptional up‐ or downregulation, but it is also influenced by the activity of neighboring pathways that produce—or compete for—shared metabolite pools. Therefore, we investigated where metabolite fluxes might be redirected in pathways that produce or consume the precursors of the MVA and MEP pathways. Specifically, we analyzed changes in gene expression for pathways that utilize the key metabolite acetyl‐CoA, precursors of isoprenoids in the MVA pathway, as well as the biosynthesis of fatty acids in the chloroplast, of triacylglycerols (TAGs) in the endoplasmic reticulum (ER), and of the tricarboxylic acid cycle (TCA) cycle in the mitochondrion, as well as glycolysis and gluconeogenesis pathways that can produce or consume pyruvate or glyceraldehyde 3‐phosphate (G3P). Additionally, we examined the Calvin–Benson (CB) cycle, which provides another crucial metabolite, G3P, the direct precursor for the MEP pathway.

We observed a marked upregulation of the genes encoding the TCA cycle enzymes. Surprisingly, despite the low photosynthetic activity observed in previous experiments (Figure 1b), the CB cycle was partially upregulated. Moreover, all glycolytic pathways, including the pentose phosphate/phosphoketolase pathway in the cytosol and the Entner–Doudoroff pathway in the chloroplast (Fabris et al., 2012; Huang et al., 2024), also showed upregulation (Figure 2b). Even though some genes related to glycolysis/gluconeogenesis showed downregulation, all enzymes of all rate‐limiting steps of glycolysis were upregulated, including the glucokinase (*Phatr3_J15495*) that initiates the first step of the pathway and two out of three genes (*Phatr3_J14284*, *Phatr3_EG02209*) annotated as 6‐phosphofructokinase, which is recognized as the major limiting step in other organisms (Zuo et al., 2021). Furthermore, five out of seven genes annotated as encoding pyruvate kinases (*Phat3_J22404*, *Phat3_J49089*, *Phat3_J45997*, *Phat3_J46001*, *Phat3_J22913*), another rate‐limiting step, were upregulated, while the other two were not differentially regulated. This upregulation of glycolytic pathways, along with the TCA cycle, suggests that pyruvate produced via glycolysis could be converted to acetyl‐CoA in the mitochondria, fueling the TCA cycle. Notably, this differential regulation of the TCA cycle was not observed in shorter phosphate depletion experiments (Matthijs et al., 2017), with even down‐regulation of the cycle having been reported in a 2‐day phosphate depletion experiment (Feng et al., 2015). This implies that the TCA cycle upregulation may be a physiological response specific to later stages of phosphate depletion. Additionally, the enhanced activity of glycolytic pathways likely increases the carbon flux toward TAG synthesis by providing key intermediates like dihydroxyacetone phosphate (DHAP), the precursor of glycerol‐3‐phosphate, and pyruvate, the precursor of acetyl‐CoA. A similar induction of glycolysis, gluconeogenesis, and the pentose phosphate pathway was observed in an earlier study after 2–3 days of phosphate depletion (Alipanah et al., 2018). Overall, nucleic acid degradation, which frees ribose‐5‐phosphate for further catabolism into TAG precursors while simultaneously releasing phosphate and reducing equivalents, may represent a beneficial adaptive strategy during phosphate scarcity.

Most genes in *Phaeodactylum tricornutum* have not been functionally and experimentally characterized. Functions are often predicted based on sequence homology with other organisms. This can lead to uncertainties about which genes to include in a pathway when analyzing transcriptional regulation of pathways. The highest upregulated genes of the CB cycle, for instance, were three fructose bisphosphate aldolases (*Phatr3_J23247*, *Phatr3_J29014*, and *Phatr3_J42447*). However, these enzymes have not been characterized and could also be part of glycolytic pathways, unrelated to carbon fixation. The only downregulated gene in the TCA cycle, as defined by Ding et al. (2023), was *Phatr3_J20934*. This enzyme is annotated as 3‐isopropylmalate dehydrogenase, which is not involved in the TCA cycle. This highlights the need for further functional characterization of the proteome of *P. tricornutum*.

*Phaeodactylum tricornutum* is known to synthesize and accumulate lipids in the absence of phosphate (Abida et al., 2014; Cruz de Carvalho et al., 2016; Huang et al., 2019). However, we observed a downregulation of fatty acid biosynthesis. For example, one of the rate‐limiting steps, the enzyme acetyl‐CoA carboxylase (*Phatr3_J55209*), which converts acetyl‐CoA to the main fatty acid building block malonyl‐CoA, showed the strongest down‐regulation. This down‐regulation of lipid biosynthesis accompanied by enhanced lipid production seems counterintuitive, but this transcriptome–metabolome discrepancy is not entirely new in the context of nutrient stress in *P. tricornutum*. Lipid accumulation during nitrogen depletion, for instance, was previously observed to contrast with the downregulation of several lipid biosynthesis genes (Matthijs et al., 2017). It is important to emphasize that it was observed that particularly under prolonged phosphate depletion, *P. tricornutum* produces long intergenic nonprotein coding RNAs (lincRNAs) that often might influence the transcription of their complementary mRNA (Cruz de Carvalho et al., 2016). The regulatory effect of such lincRNAs might potentially result in such nonintuitive transcriptome–metabolome discrepancies.

Overall, we confirmed trends by our RNA‐seq analysis, trends in terpenoid metabolic network remodeling that we had observed in previously published datasets (Cruz de Carvalho et al., 2016). We showed vast downregulation of the plastidial MEP and pigment biosynthesis pathways and an upregulation of the sterol pathway. A complete list of genes defined for each pathway, based on previous studies (Ding et al., 2023; Fabris et al., 2014, 2020), is in Table S3.

### Prolonged phosphate depletion does not affect total sterol accumulation but impairs pigment biosynthesis

To assess whether the transcriptional remodeling of the aforementioned pathways resulted in the differential accumulation of endpoint terpenoid metabolites, such as pigments and sterols, we profiled and quantified them from diatom biomass derived from the same experiment.

**FIGURE 3:**
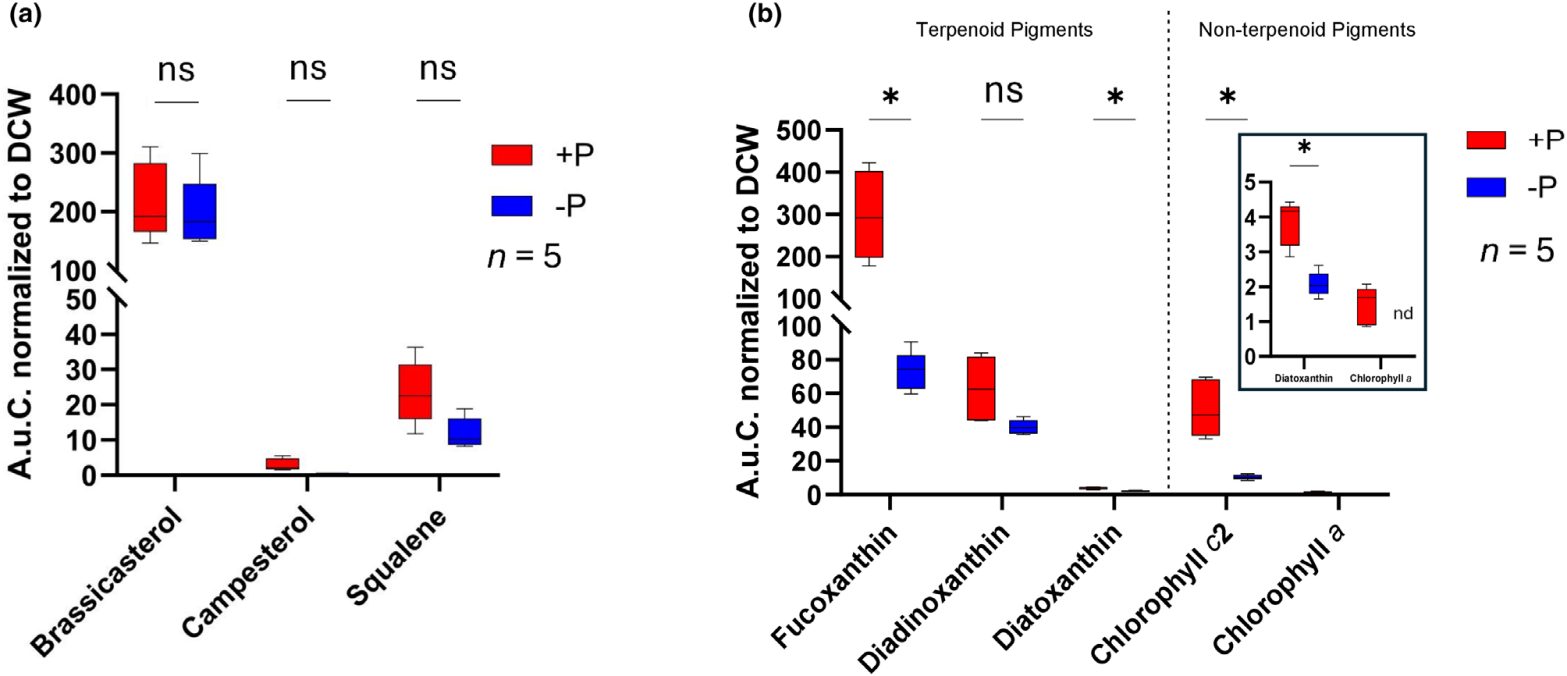
Analysis of terpenoid biosynthesis and accumulation during prolonged phosphate depletion in *Phaeodactylum tricornutum*. (a) Comparison of the main sterols in *P. tricornutum*, brassicasterol, campesterol, and their precursor squalene, normalized to dry cell weight (DCW) under +P and ‐P conditions. (b) Comparison of major pigments in *P. tricornutum*, fucoxanthin, chlorophyll (chl) *c*2, chlorophyll *a* (chl *a*), diadinoxanthin, and diatoxanthin, normalized to DCW under +P and ‐P conditions (*n* = 5). The box shows the abundance of the lowly accumulated pigments diatoxanthin and chl *a* in detail. The relative amount of pigment produced is indicated by the area under the curve (A.u.C.) of the respective peak measured by the high‐performance liquid chromatography (HPLC)‐diode array detection (DAD) and is normalized to the DCW. Differences in means between groups were calculated by two‐tailed Student's *t*‐test. Asterisks (*) indicate significant differences (*p* < 0.05) between +P and −P conditions, and “ns” indicates no statistically significant difference could be detected and “nd” indicates nondetectable, as the content was below measurable levels.

In contrast to the observed upregulation of the sterol pathway, we could not detect any differential accumulation of the main sterols, brassicasterol, campesterol, and their key pathway precursor squalene, during prolonged phosphate depletion (Figure 3a). This discrepancy between transcriptional upregulation and the lack of metabolite accumulation is intriguing and suggests that post‐transcriptional or post‐translational regulatory mechanisms might be at play. This could potentially be mediated by lincRNAs, as has been described by Cruz de Carvalho et al. (2016). Alternatively, this could be due to an enhanced flux of acetyl‐CoA toward lipids, which are known to accumulate in *Phaeodactylum tricornutum* during phosphate depletion (Cruz de Carvalho et al., 2016; Huang et al., 2019) even though lipid metabolism showed no clear transcriptional upregulation in our prolonged phosphate depletion experiment. In contrast, we could observe upregulation in the TCA cycle genes, which also feed on acetyl‐CoA (Figure 2b). The major question of why the upregulation of the sterol pathway does not result in higher sterol production could also be explained by the presence of sterol‐responsive element‐binding protein (SREBP)‐mediated transcriptional regulation. The SREBPs are key TFs that regulate the biosynthesis of cholesterol, fatty acids, and triglycerides and are conserved in many species, including humans (Brown & Goldstein, 1997), mice (Horton et al., 1998), yeasts (Gómez et al., 2021), fruit flies (Li et al., 2022), and nematodes (Lee et al., 2015). Their presence has been hypothesized in *P. tricornutum* (Jaramillo‐Madrid et al., 2019) but remains uninvestigated. Preliminary evidence has suggested that lower sterol levels might regulate the expression of genes encoding enzymes involved in sterol biosynthesis and upstream pathways (D'Adamo et al., 2019). Even though the decrease in sterols observed in this study during prolonged phosphate depletion was not statistically significant, we observed a consistent drop in sterol levels in phosphate‐depleted cultures (Figure 3a).

We searched for sterol‐binding TFs that have similar functional domains as human SREBP1 (Uniprot entry identifier: P36956) and for steroid acute regulatory protein (Uniprot entry identifier: P49675), respectively, in Bacillariophyta by mining this group with PSI‐Blast, but we could not find any candidate gene. This suggests that there might be no such homologous proteins in *Phaeodactylum. tricornutum*, and a direct sterol‐sensing transcription regulation—if present—might be based on different and as yet unknown mechanisms.

In contrast, a drastic decrease in pigment content could be observed in phosphate‐depleted cultures, and the content of fucoxanthin and diatoxanthin decreased 4.1‐ and 1.8‐fold, respectively. This aligns with a decrease in photosynthetic activity in phosphate‐depleted cultures and with the transcriptional down‐regulation of key MEP pathways and pigment biosynthesis genes (Figure 2a). Furthermore, nonterpenoid pigments, such as chl *c*2 decreased 4.9‐fold, while chl a decreased to nondetectable amounts (Figure 3b).

In summary, our metabolite analysis on the two main terpenoid metabolic sinks of *Phaeodactylum tricornutum* complemented our transcriptomic findings, revealing a significant reduction in key photosynthetic pigments, while sterol content remained unchanged despite the transcriptional upregulation of sterol biosynthesis genes. This emphasizes that transcriptional data do not always translate directly into pathway regulations and underlines our current lack of understanding on how these pathways are regulated.

### GPP pools increase during prolonged phosphate depletion

Although squalene and sterols serve as proxies for estimating FPP availability and carotenoids reflect the availability of GGPP, they do not necessarily indicate much about the availability of GPP. *Phaeodactylum tricornutum* accumulates substantial pools of free GPP, for which origin and fate are still elusive (Fabris et al., 2020). Dimethylallyl diphosphate and IPP, the products of the MVA pathway, are converted by prenyltransferases into GPP and FPP, eventually entering the steroid pathway via squalene (Fabris et al., 2014). Geranyl diphosphate could be an intermediate of FPP formation (like in yeast), or it could be synthesized independently (as in several plants). *Phaeodactylum tricornutum* has five genes annotated as prenyltransferases, but their subcellular localization has only been predicted computationally (Fabris et al., 2020), and their substrate/product specificity has not been determined yet. Notably, *Phatr3_49325*, one of the cytosolic prenyltransferases, was upregulated during prolonged phosphate depletion, while another cytosolic prenyltransferase, *Phatr3_47241*, was not differentially expressed.

**FIGURE 4:**
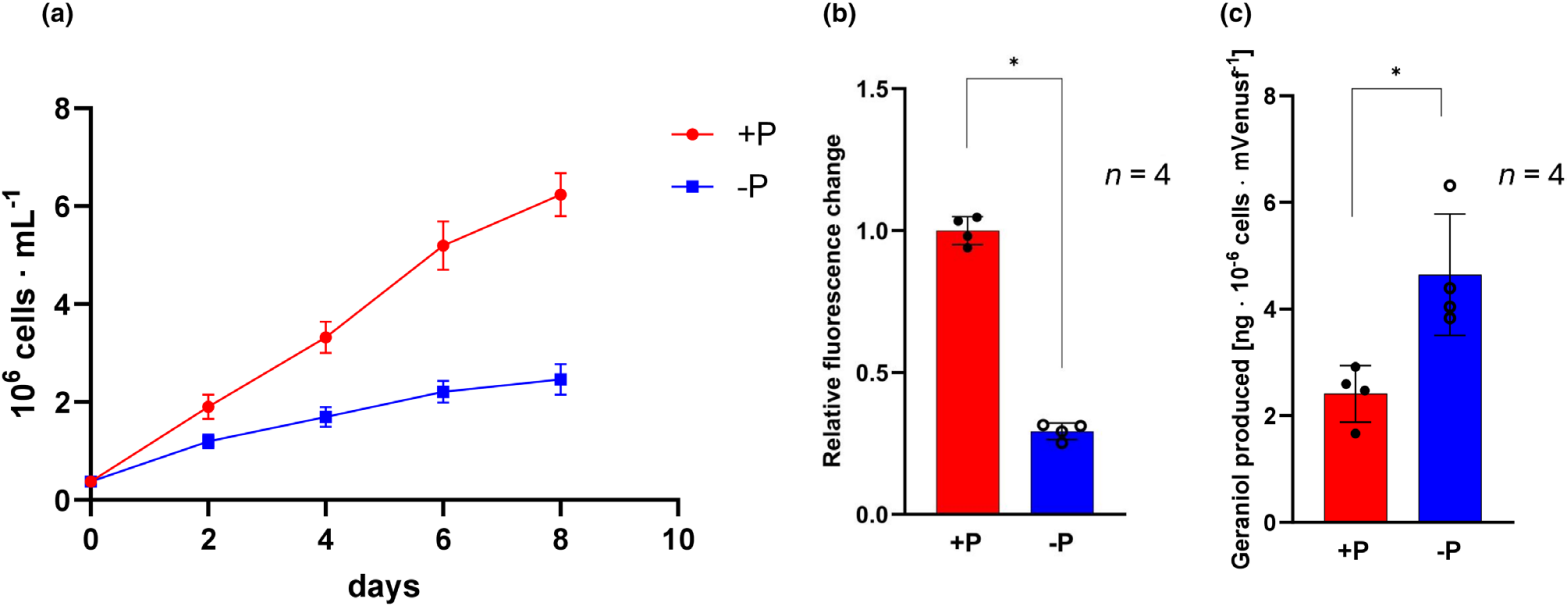
Geraniol production in prolonged phosphate depletion. (a) Growth curves of cultures expressing the geraniol synthase (GES)‐mVenus fusion protein, grown for 8 days in the presence or absence of phosphate. (b) Relative mVenus fluorescence change in phosphate‐depleted (−P) cultures compared to phosphate‐repleted (+P) cultures. (c) Geraniol production per 10^6^ cells, normalized to the mean mVenus fluorescence. Data are displayed as mean ± standard error of the mean for *n* = 4, with a significant difference evaluated by two‐tailed *t*‐tests, marked by * (*p* < 0.05).

As previously mentioned, the MVA pathway did not show a clear trend in its differential transcriptional regulation. To estimate how prolonged phosphate depletion influences the productivity of this pathway, we introduced a mVenus‐tagged GES from *Catharanthus roseus* (CrGES‐mV), targeted to the cytosol, to convert GPP into geraniol, a monoterpenoid emitted in the culture medium, which is more stable and easier to quantify than GPP. CrGES has been previously expressed and characterized in *Phaeodactylum tricornutum* (Fabris et al., 2020; George et al., 2020). In these cell lines, a full‐length plant recombinant enzyme localizes in the diatom cytosol, and phenotypes of *P. tricornutum* engineered with this construct have been shown to consistently convert part of the cytosolic pool of GPP into heterologous geraniol (Fabris et al., 2020). In using geraniol production as an indirect proxy for estimating changes in GPP levels, we assumed a linear correlation between the amount of produced geraniol and cytosolic GPP content, as well as a linear correlation between mVenus fluorescence levels of cells and intracellular protein levels of CrGES‐mVenus. Since the phenotypes have been thoroughly characterized previously, we focused our experiment on a single representative cell line expressing the transgenic construct (Figure 4).

After 8 days of cultivation in ESAW with and without phosphate, we observed a general decrease in mVenus fluorescence levels under phosphate‐starved conditions, measured with flow cytometry, indicating lower expression or availability of the GES‐mV fusion enzyme (Figure 4b). Phosphate‐starved cultures accumulated only about half the geraniol amount compared to cultures with phosphate. However, once normalized with the lower content of GES‐mV, we estimated an almost two‐fold increase in geraniol yield in phosphate‐depleted cultures (Figure 4c, two‐tailed Students *t*‐test, *p*‐value = 0.012, *α* = 0.05, *df* = 6). This suggests that cells might increase their cytosolic GPP pool by nearly two‐fold during phosphate depletion.

It is counterintuitive that these elevated intermediate prenyl levels did not result in a higher accumulation of sterols downstream. In combination with the observation that squalene levels did not increase during phosphate depletion, it suggests that somewhere between these two metabolites there might be a regulative bottleneck step, gatekeeping the flux either at the level of FPP or PSDP. The biosynthesis and accumulation of GPP in free cytosolic pools is a peculiar aspect of *Phaeodactylum tricornutum* metabolism. These results provide extra elements to help us understand the role of GPP in diatoms: The fluctuation of GPP pools suggests that GPP might not be involved in the conversion to FPP and channeled into the biosynthesis of sterols but be available for other functions or conversions. In diatom species that produce GPP‐derived toxins such as domoic acid (Brunson et al., 2018), the upregulation of GPP biosynthesis and the transcriptional reprogramming of terpenoid biosynthesis in prolonged nutrient stress provide new elements for understanding mechanisms at the basis of the onset of toxin production (Cochlan et al., 2023).

The nonintuitive changes in the terpenoid metabolism during phosphate depletion could be advantageous for achieving higher production titers in a biotechnological context. Specifically, the drastic increase in GPP levels could boost production of monoterpenoids and high‐value derivatives (Fabris et al., 2020). However, it is crucial to use the right promoter in combination with this culturing condition to avoid the sharp drop in protein content observed in our experiments using the *Phatr3_J49202* promoter for GES expression (Fabris et al., 2020). Using a phosphate depletion‐inducible promoter like the alkaline phosphatase (*Phatr3_J49678*) promoter (p*AP1*), cells could be grown without energy investment into heterologous protein expression until the monoterpenoid synthase of interest is expressed when phosphate becomes limiting and pools rise. In fact, GES has previously been expressed under p*AP1* (Fabris et al., 2020; George et al., 2020), but geraniol production under this promoter has not been compared to the production under constitutive promoters such as in this study. Testing whether the approach of using a promoter such as p*AP1* would indeed result in higher titers than when using a constitutive promoter such as p*Phatr3_J49202* was out of the scope of this work and remains to be tested.

Our estimation approach was simplified by assuming a linear relationship between enzymatic conversion rates and educt concentration, which does not accurately reflect the enzyme kinetics described by Michaelis and Menten (Michaelis et al., 2011). However, since GPP is an intermediate in the pathway and likely does not accumulate in large quantities, we considered a linear correlation between changes in educt and product concentrations to be a reasonable approximation. This is because the initial part of the Michaelis–Menten curve resembles a linear relationship between substrate concentration and reaction velocity.

### Candidate TFs may be involved in the regulation of terpenoid biosynthesis in prolonged phosphate starvation

The observation of a concerted differential regulation of terpenoid pathways in prolonged phosphate depletion, especially among key sterol biosynthesis genes, led us to hypothesize that there could exist common regulators orchestrating this effect. We therefore set out to search for TFs that could be involved in this mechanism. To investigate potential TFs regulating terpenoid biosynthesis in *Phaeodactylum tricornutum* during phosphate starvation, we searched the regions (600 bp) upstream of the start codons from upregulated terpenoid biosynthesis genes for conserved nucleotide sequences (Table S4), with MEME (Bailey et al., 2015). This analysis revealed a significantly enriched 9 bp motif (MEME enrichment analysis, E‐value < 0.05, Figure S3a). A complete list of the submitted promoter sequences and the number of occurrences of the discovered motif in the respective putative promoter is provided in Table S4.

Subsequently, we used the TOMTOM (Bailey et al., 2015) tool within the MEME Suite to search for known TFs that might bind to this motif in the *Phaeodactylum tricornutum* database. This search yielded a match to the TF candidate *Phatr3_J48955*, which belongs to the cysteine‐rich polycomb‐like protein (CPP) family (Figure S3b). Notably, *Phatr3_J48955* is co‐regulated with *PtTryp2*, a key regulator of phosphate starvation, as reported by You et al. (2022), and has a binding site in its promoter for *PtPSR*, the main phosphate starvation response TF.

In the PhaeoNet data set (Ait‐Mohamed et al., 2020), genes of *Phaeodactylum tricornutum* were subjected to co‐expression analyses and clustered into 28 co‐regulated modules, named after colors, based on their expression changes in different environmental conditions. We hypothesized that genes that are present in the same module and are functionally connected might be regulated by a common TF. Genes in the MEP pathway are predominantly co‐regulated with the lightsteelblue1 module in the PhaeoNet dataset, while those in the carotenoid biosynthesis pathway are mostly associated with the blue and pale turquoise modules (Figure S4). The MVA pathway and sterol biosynthesis genes are primarily co‐regulated with the dark gray module (Figure S4a). Interestingly, the CPP TF (*Phatr3_J48955*) was co‐regulated within the upregulated sterol biosynthesis pathway genes in the dark gray module. This indicates that *Phatr3_J48955* could be a good TF candidate to link the PtPSR–SPX–PtTryp2 phosphate starvation response cascade to the upregulation of the sterol pathway.

To further narrow down TF candidates, we clustered the TF list from Zhou et al. (2022) into their respective PhaeoNet co‐regulation modules (Table S5). Notably, only three TFs from Zhou et al.'s list were co‐regulated with the lightsteelblue1 module, which governs the MEP pathway. These included *Aureo1b* (*Phatr3_J15977*), *Sigma70.4* (*Phatr3_J9312*), and *Phatr3_J50411*, the two latter down‐regulated in our phosphate starvation expression data set. It is likely that one or more of these factors are involved in the regulation of the MEP pathway. However, two known regulators of the MEP and pigment biosynthesis pathways, *HSF1* (*Phatr3_J49566*; Song et al., 2023) and *HSF3* (*Phatr3_J44200*; Zhao et al., 2024), were co‐regulated within the cyan module, which does not contain MEP or carotenoid biosynthesis pathway genes.

In conclusion, we identified three strong candidate TFs that might regulate the MEP pathway in a phosphate‐independent manner. These findings indicate potential TF candidates that regulate terpenoid biosynthesis under nutrient stress in *Phaeodactylum tricornutum*.

## CONCLUSIONS

This study has provided a comprehensive overview of how the terpenoid metabolic network is regulated in the diatom *Phaeodactylum tricornutum* during prolonged phosphate depletion, which is one of the few conditions identified so far as to be able to transcriptionally upregulate the diatom sterol metabolism. In this environmental condition, *P. tricornutum* shifts its metabolism away from pigment production while maintaining sterol homeostasis. Despite the transcriptional upregulation of the sterol biosynthesis pathway, increased sterol accumulation was not observed, highlighting discrepancies between the transcriptome and the metabolic phenotype. Another intriguing finding was the accumulation of GPP during phosphate depletion without a downstream increase in sterol levels. This raised questions about the potential, yet undiscovered, roles of GPP in diatom metabolism, which is particularly relevant in species known to produce GPP‐derived domoic acid, such as *Pseudo‐nitzchia* spp. The possibility that phosphate depletion induced GPP accumulation could have biotechnological applications also requires further exploration. Additionally, the mechanisms by which sterol levels are sensed and how this feedback regulates the expression of sterol biosynthesis genes remain to be resolved. In this study, we identified TF candidates that may specifically regulate the MEP pathway and sterol biosynthesis. Further functional characterization of these TFs will be essential for unraveling the regulatory mechanisms underlying terpenoid metabolism in diatoms.

Our work provides new elements for understanding the complex and unexplored dynamic of the metabolism of terpenoid in diatoms and also highlights several substantial knowledge gaps. Our limited understanding of many genes in *Phaedactylum tricornutum* continues to hinder the full elucidation of the terpenoid metabolic network. Further characterization of these genes is critical for advancing our knowledge of terpenoid metabolism in diatoms, and such investigation is currently ongoing in our research group. Addressing these gaps will be crucial for both understanding the ecological roles of terpenoids in diatoms and harnessing their potential for biotechnological applications.

## AUTHOR CONTRIBUTIONS

**Florian Pruckner:** Conceptualization (supporting); data curation (lead); formal analysis (lead); investigation (lead); methodology (lead); visualization (lead); writing – original draft (lead); writing – review and editing (lead). **Luca Morelli:** Formal analysis (supporting); investigation (supporting); methodology (supporting); writing – review and editing (supporting). **Payal Patwari:** Investigation (supporting); methodology (supporting); writing – review and editing (supporting). **Michele Fabris:** Conceptualization (lead); formal analysis (supporting); funding acquisition (lead); methodology (supporting); project administration (lead); resources (lead); supervision (lead); writing – review and editing (lead).

## Supporting information


**Figure S1.** (a) Hierarchical clustering and (b) principal component analysis of samples generated in the prolonged phosphate depletion experiment (*n* = 5).

**Figure S2.** Venn diagram showing the comparison of overall differentially expressed genes (DEGs) identified in this study (blue) and those reported by Cruz de Carvalho et al. (2016; green) during prolonged phosphate starvation. The overlap region indicates the number of DEGs common to both studies. (a) Overall DEGs, (b) upregulated DEGs, and (c) downregulated DEGs.

**Figure S3.** Analysis of putative promoter regions of terpenoid genes upregulated during phosphate starvation, via the online tool MEME suit. (a) Discovered conserved motif among the submitted promoter (600 bp upstream of start codon). (b) The discovered motif significantly (*p* < 0.05) aligns to the binding motif of *Phatr3_J48955*, as predicted by TOMTOM analyses.

**Figure S4.** (a) Distribution of genes involved in methylerythritol 4‐phosphate (MEP), carotenoid biosynthesis, mevalonate (MVA), and sterol biosynthesis pathways into different co‐regulation PhaeoNet modules as defined by Ait‐Mohamed et al. (2020). (b) Differentially expressed transcription factors in the main modules associated with the MEP (lightsteelblue1), pigment biosynthesis (blue, pale turquoise), MVA (dark gray), and sterol biosynthesis pathway (dark gray). Phosphate starvation response regulators are associated with the brown4 module.

**Table S1.** Expression matrix of the ribonucleic acid (RNA)‐seq experiment of this study.

**Table S2.** Gene orthology (GO) analysis of up‐ and down‐regulated differentially expressed gene (DEG) during prolonged phosphate depletion (Wald test, Benjamini–Hochberg adjusted *p* < 0.01, log_2_FC >1.5). Analysis was performed via g:profiler (https://biit.cs.ut.ee/gprofiler/gost). Multiple testing corrections were carried out with the Benjamin–Hochberg method with a significance threshold of 0.05. Only the top 12 hits are shown.

**Table S3.** Genes associated with different metabolic pathways, based on previously published studies.

**Table S4.** Promoters analyzed with MEME.

**Table S5.** Predicted transcription factors list with their associated PhaeoNet mixed modules.

## Data Availability

The RNA‐seq data generated in this study have been deposited in the European Nucleotide Archive (ENA) under the study accession number PRJEB80375.
