# Supplementary figures and images for: Remodeling of the terpenoid metabolism during prolonged phosphate depletion in the marine diatom Phaeodactylum tricornutum
Source: J Phycol. 2025 Apr 15;61(3):512–28. doi: 10.1111/jpy.70014 (PMC12168105; doi:10.1111/jpy.70014)

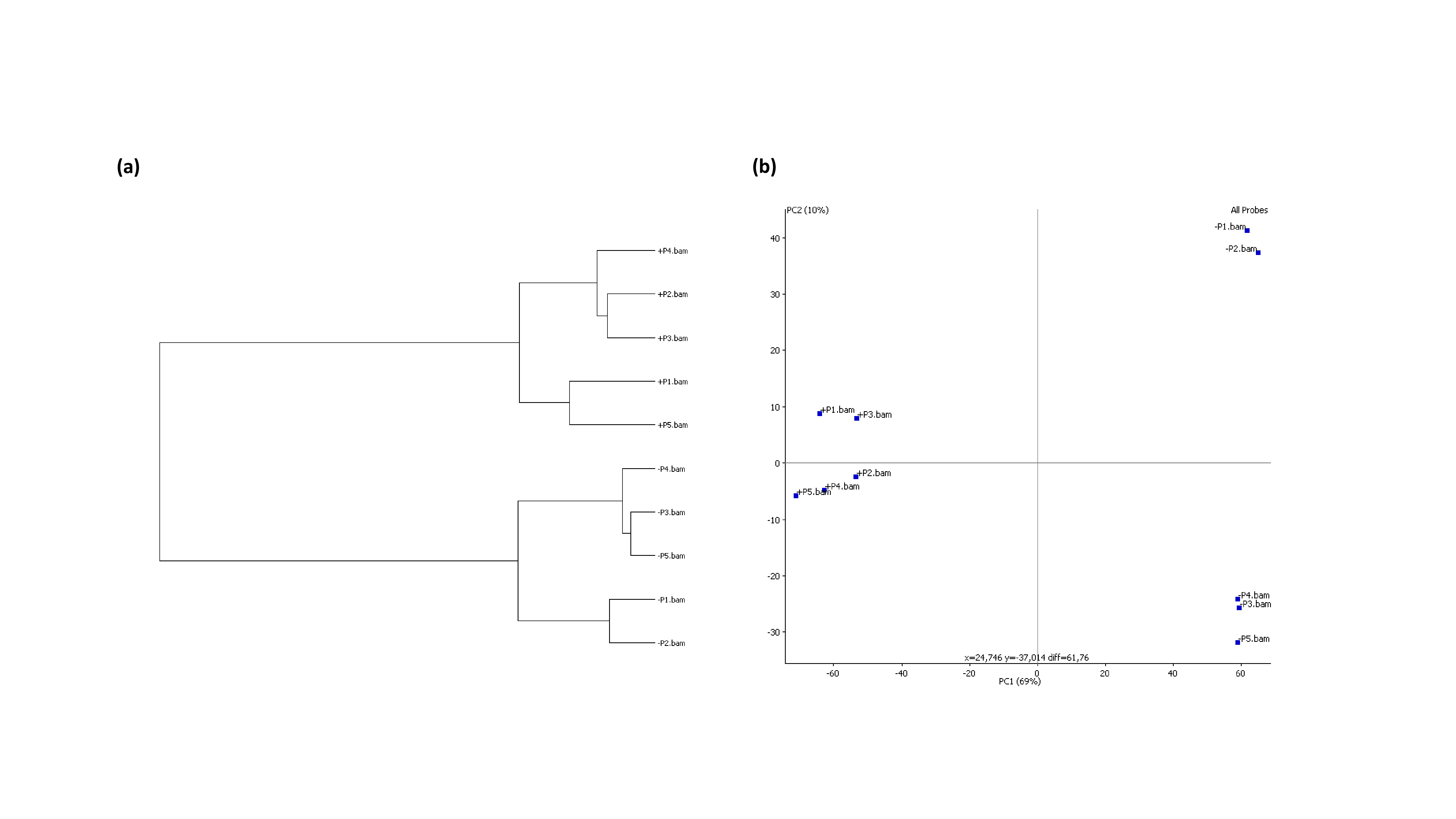

Supplement: Supplementary file 1 — Figure S1. (a) Hierarchical clustering and (b) principal component analysis of samples generated in the prolonged phosphate depletion experiment (n = 5). [file JPY-61-512-s008.tiff]

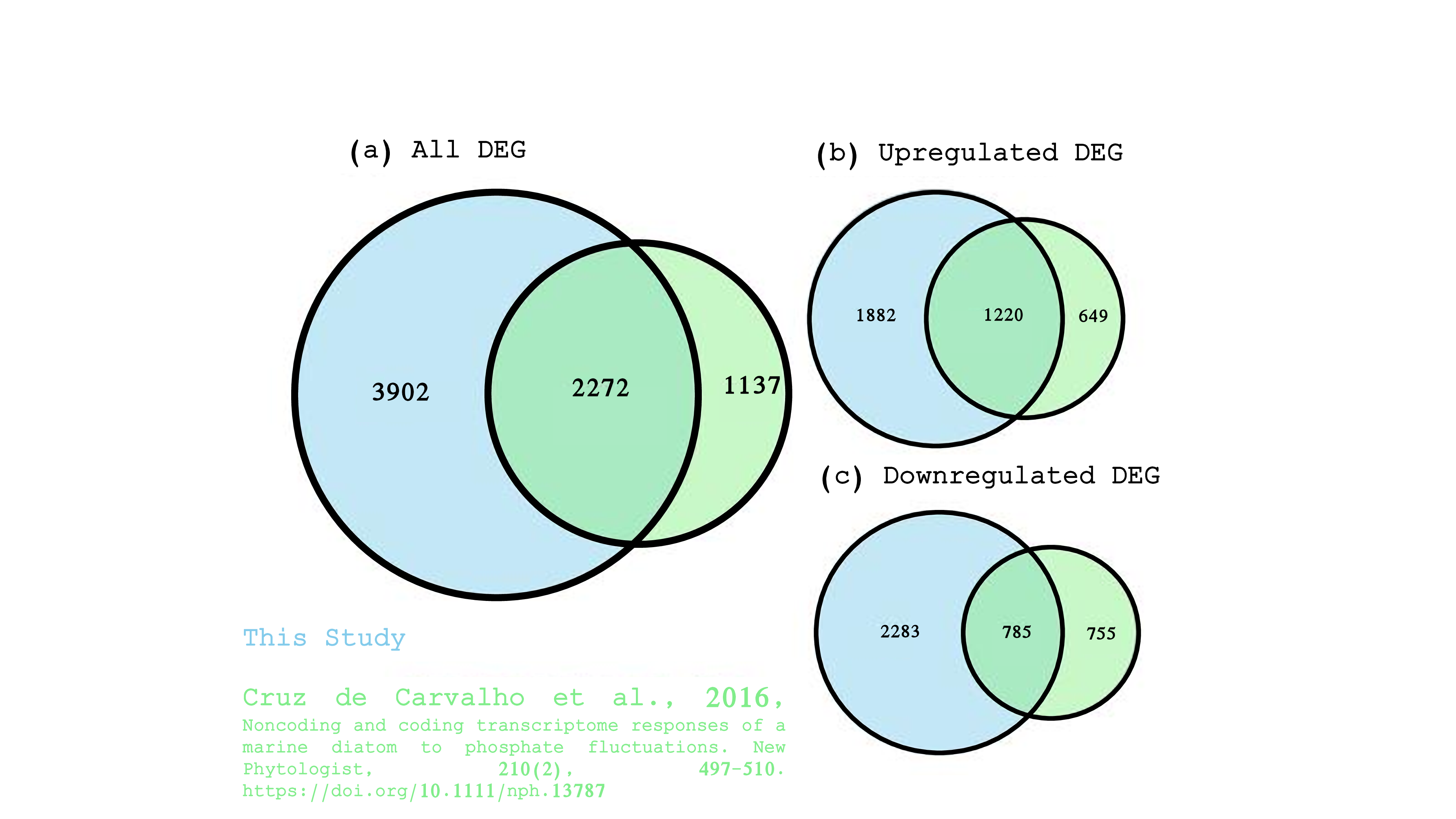

Supplement: Supplementary file 2 — Figure S2. Venn diagram showing the comparison of overall differentially expressed genes (DEGs) identified in this study (blue) and those reported by Cruz de Carvalho et al. (2016; green) during prolonged phosphate starvation. The overlap region indicates the number of DEGs common to both studies. (a) Overall DEGs, (b) upregulated DEGs, and (c) downregulated DEGs. [file JPY-61-512-s001.tiff]

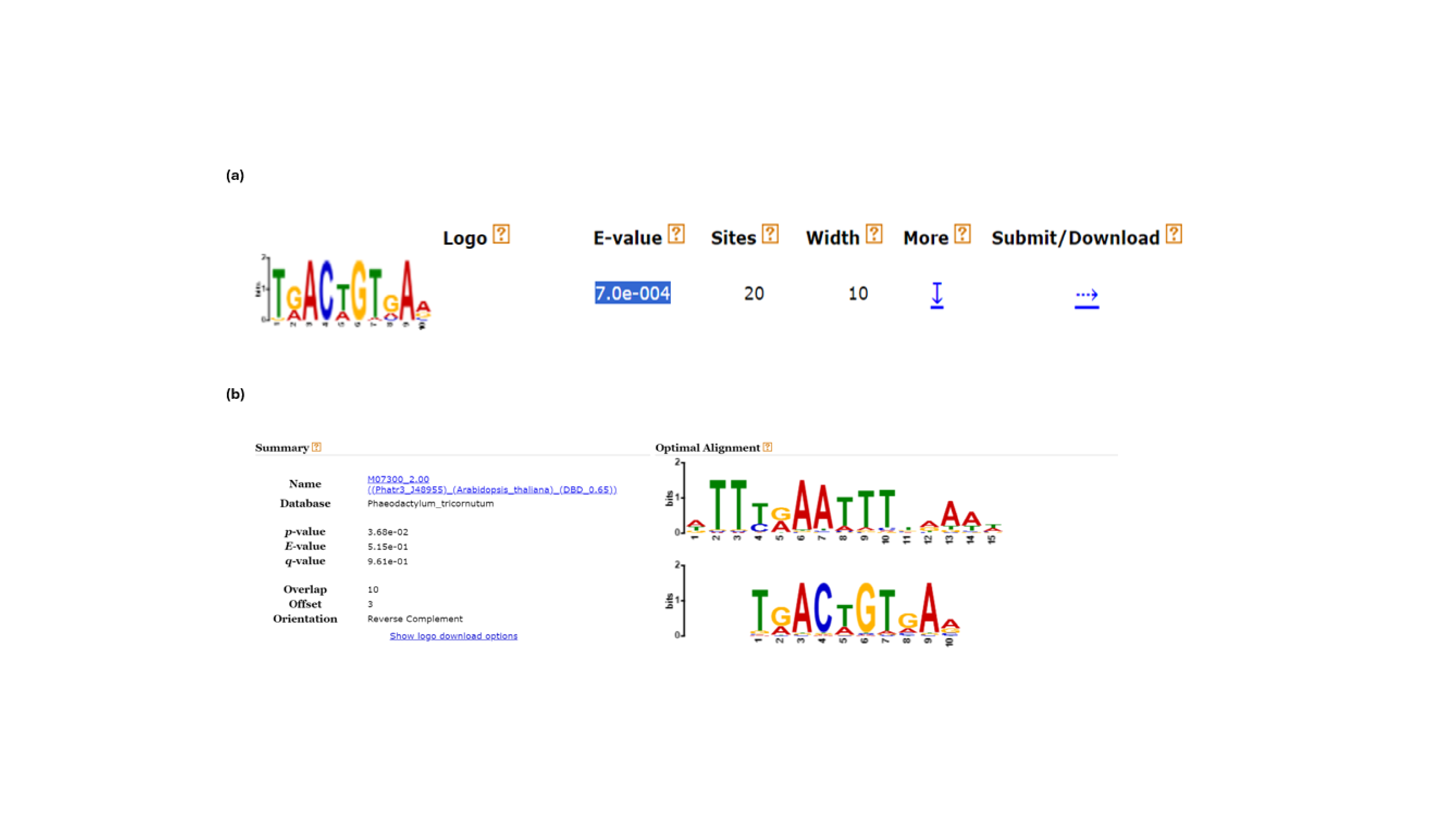

Supplement: Supplementary file 3 — Figure S3. Analysis of putative promoter regions of terpenoid genes upregulated during phosphate starvation, via the online tool MEME suit. (a) Discovered conserved motif among the submitted promoter (600 bp upstream of start codon). (b) The discovered motif significantly (p < 0.05) aligns to the binding motif of Phatr3_J48955, as predicted by TOMTOM analyses. [file JPY-61-512-s004.tiff]

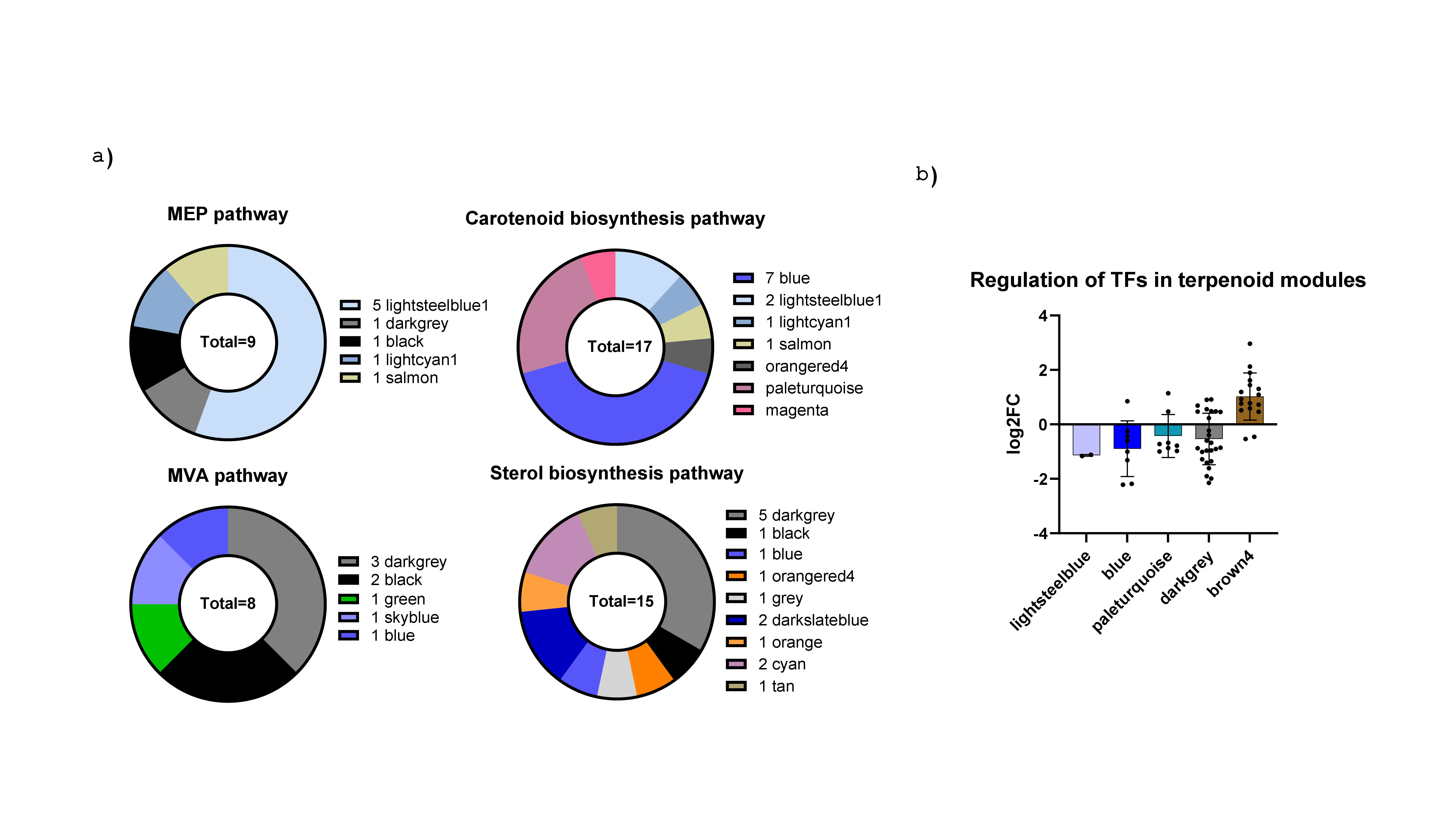

Supplement: Supplementary file 4 — Figure S4. (a) Distribution of genes involved in methylerythritol 4‐phosphate (MEP), carotenoid biosynthesis, mevalonate (MVA), and sterol biosynthesis pathways into different co‐regulation PhaeoNet modules as defined by Ait‐Mohamed et al. (2020). (b) Differentially expressed transcription factors in the main modules associated with the MEP (lightsteelblue1), pigment biosynthesis (blue, pale turquoise), MVA (dark gray), and sterol biosynthesis pathway (dark gray). Phosphate starvation response regulators are associated with the brown4 module. [file JPY-61-512-s002.tiff]
